# Exogenous l-Valine Promotes Phagocytosis to Kill Multidrug-Resistant Bacterial Pathogens

**DOI:** 10.3389/fimmu.2017.00207

**Published:** 2017-03-06

**Authors:** Xin-hai Chen, Shi-rao Liu, Bo Peng, Dan Li, Zhi-xue Cheng, Jia-xin Zhu, Song Zhang, Yu-ming Peng, Hui Li, Tian-tuo Zhang, Xuan-xian Peng

**Affiliations:** ^1^Center for Proteomics, State Key Laboratory of Bio-Control, School of Life Sciences, Guangdong Province Key Laboratory for Pharmaceutical Functional Genes, Sun Yat-sen University, Guangzhou, China; ^2^Third Affiliated Hospital of Sun Yat-sen University, Guangzhou, China

**Keywords:** l-valine, nitric oxide, l-arginine, phagocytosis, bacterial infections

## Abstract

The emergence of multidrug-resistant bacteria presents a severe threat to public health and causes extensive losses in livestock husbandry and aquaculture. Effective strategies to control such infections are in high demand. Enhancing host immunity is an ideal strategy with fewer side effects than antibiotics. To explore metabolite candidates, we applied a metabolomics approach to investigate the metabolic profiles of mice after *Klebsiella pneumoniae* infection. Compared with the mice that died from *K. pneumoniae* infection, mice that survived the infection displayed elevated levels of l-valine. Our analysis showed that l-valine increased macrophage phagocytosis, thereby reducing the load of pathogens; this effect was not only limited to *K. pneumoniae* but also included *Escherichia coli* clinical isolates in infected tissues. Two mechanisms are involved in this process: l-valine activating the PI3K/Akt1 pathway and promoting NO production through the inhibition of arginase activity. The NO precursor l-arginine is necessary for l-valine-stimulated macrophage phagocytosis. The valine-arginine combination therapy effectively killed *K. pneumoniae* and exerted similar effects in other Gram-negative (*E. coli* and *Pseudomonas aeruginosa*) and Gram-positive (*Staphylococcus aureus*) bacteria. Our study extends the role of metabolism in innate immunity and develops the possibility of employing the metabolic modulator-mediated innate immunity as a therapy for bacterial infections.

## Introduction

Multidrug-resistant bacterial pathogens, which are unresponsive to antibiotics, pose a substantial challenge to human health and animal husbandry. Currently, Gram-negative bacteria with extended-spectrum beta-lactamases (ESBLs), methicillin-resistant *Staphylococcus aureus* (MRSA), and *Mycobacterium tuberculosis* are the top three harmful pathogens around the world that can hardly be eliminated due to multidrug resistance (1, 2). Among these Gram-negative bacteria with ESBLs, *Klebsiella pneumoniae* is a typical pathogen with high emergence that frequently promote empirical therapy failures (3, 4). Therefore, new approaches to treat such infections in the clinic are urgently needed. One possible approach would be to enhance the innate immune response of the infected host, which would restore the defense ability to kill the bacterial pathogen in a relatively risk-free manner (5).

Several lines of evidence have demonstrated that bacterial infections cause host metabolic changes, including central carbon metabolism, amino acid metabolism, and fatty acid metabolism (6–10). Pathogens also shift their metabolic programs to adapt to their new environment. More importantly, it has been demonstrated that several metabolites can be immunoregulators that modulate the function of immune cells (11–23). Examples of such metabolites include l-valine, which regulates the maturation and function of monocyte-derived dendritic cells (DCs) through a nutrient-sensitive signaling pathway (16). These results indicate that modulation of host innate immunity by metabolites may be a new valuable solution against bacterial pathogens.

Metabolomics is a powerful tool for studying metabolic processes, identifying crucial biomarkers responsible for metabolic characteristics, and revealing metabolic mechanisms. Furthermore, crucial biomarkers can be used to reprogram a metabolome, leading to a specific metabolome to cope with changes in internal and external environments (23). Using this approach, we have identified crucial biomarkers that contribute to metabolic mechanisms in bacteria and hosts in response to antibiotics and pathogen invasion. The use of these key biomarkers reprograms the bacterial and host metabolomes to eliminate bacterial resistance to antibiotics and enhances host immunity against bacterial infections, respectively (24–32).

Here, we report the use of gas chromatography–mass spectrometry (GC-MS) combined with multivariate statistical tools to characterize the blood metabolome from BALB/c mice infected by sublethal doses of *K. pneumoniae*. Furthermore, we identified a potential immunomodulatory metabolite, l-valine, which is capable of enhancing host immunity against *K. pneumoniae* infection. We were specifically interested in understanding the metabolic mechanism by which this potential compound modulates the survival-related metabolome to enhance cell anti-infective abilities. The results are reported as follows.

## Materials and Methods

### Ethics Statement

All work was conducted in strict accordance with the recommendations in the Guide for the Care and Use of Laboratory Animals of the National Institutes of Health. The protocol was approved by the Institutional Animal Care and Use Committee of Sun Yat-sen University (Animal Welfare Assurance Number: I6).

### Chemicals

Fluorescein isothiocyanate (FITC, F7250), l-valine (V0513), l-arginine (A8094), lipopolysaccharide (LPS) (LPS, L4524), and *S*-(2-boronoethyl)-l-cysteine (SML184, arginase inhibitor) were purchased from Sigma-Aldrich. LY294002 was purchased from KeyGen Biotech, China. Two antibodies, phospho-Akt1-S473 (AP0140) and β-actin (AC004), were from Abclonal, USA. Four nitric oxide (NO) inhibitors, Carboxy-PTIO (PTIO), l-NMMA, SMT, and l-NAME, were purchased from Beyotime Biotechnology, China. The urea assay kit and Nitric Oxide Assay Kit were purchased from BioVision (Mountain View, CA, USA) and Beyotime (Beijing, China), respectively. LY294002 was dissolved in DMSO. Meanwhile, the equal volume of DMSO was also added in the other groups of this experiment as solvent control to exclude the effects of DMSO on phagocytosis.

### Bacterial Strains, Culture Conditions, and Experimental Animals

All bacterial species in this study were obtained from the collection maintained at our laboratory. The bacterial strains used in the present study consisted of the following clinical isolates: *K. pneumoniae* (No. 0367 and No. 1924), *Escherichia coli*, MRSA, and *Pseudomonas aeruginosa*. The *E. coli* MCC-5 and HCC-13 were isolated from chickens, and the other bacteria were isolated from humans. The bacterial strains were cultured from frozen stocks in LB medium in a shaker bath at 37°C. Bacterial cells from overnight cultures were diluted 1:100 into 100 mL of LB medium. The cultures were harvested at an absorbance of 1.0 (OD600) by centrifugation at 7,000 rpm for 15 min at 4°C. The cells were washed in 40 mL of sterile saline (0.85% NaCl) and then resuspended in 0.85% NaCl. Male mice (BALB/c, pathogen-free), weighing 24 ± 2 g from the same litters and obtained from the Animal Center of Sun Yat-sen University, were reared in cages fed with sterile water and dry pellet diets. Between 50 and 100 μL blood was obtained from the orbital vein of each mouse as the non-infection group. Then, each mouse was intraperitoneally or intravenously infected by inoculation with the indicated colony-forming units (CFUs) of bacteria. Equal amounts of blood were collected from each mouse in the experimental group at 6 h post-infection using the same approach as for before infection. The experimental group was further divided into the dead and survival groups at 15 days depending upon whether the mice either succumbed to the infection or survived after infection.

### Metabolite Extraction in Mouse Plasma

Total metabolites extracted from plasma were performed as described previously (27). Briefly, 50 μL plasma was quenched by using 50 μL cold methanol and collected by centrifugation at 8,000 rpm for 3 min. This step was repeated two times. The two supernatants were mixed, and aliquot of sample was transferred to a GC sampling vial containing 5 μL 0.1 mg mL^−1^ ribitol (Sigma) as an analytical internal standard and then dried in a vacuum centrifuge concentrator before the subsequent derivatization. Two technical replicates were prepared for each sample.

### Derivatization and GC-MS Analysis

Samples must be derived before GC-MS analysis. Therefore, 80 μL of methoxamine/pyridine hydrochloride (20 mg mL^−1^) was added to dried samples to induce oximation for 1.5 h at 37°C, and then 80 μL of the derivatization reagent MSTFA (Sigma) was mixed and reacted with the sample for 0.5 h at 37°C. A 1 μL aliquot of the derivative of the supernatant was added to a tube and analyzed using GC-MS (Trace DSQ II, Thermo Scientific). The separation conditions of GC-MS consisted of an initial temperature of 70°C (5 min) with a uniform increase to 270°C at a speed of 2°C min^−1^ (5 min); 0.5 μL sample volume, splitless injection; injection temperature, 270°C; interface temperature, 270°C; ion source (EI) temperature, 30°C; ionization voltage, 70 eV; quadrupole temperature, 150°C; carrier gas, highly pure helium; velocity, 1.0 mL min^−1^; and full scan way, 60–600 *m*/*z*.

In data processing, spectral deconvolution and calibration were performed using AMDIS and internal standards. A retention time (RT) correction was performed for all the samples, and then the RT was used as reference against which the remaining spectra were queried and a file containing the abundance information for each metabolite in all the samples was assembled. Metabolites from the GC-MS spectra were identified by searching in National Institute of Standards and Technology (NIST) library used the NIST MS search 2.0. The resulting data matrix was normalized using the concentrations of added internal standards that were subsequently removed so that the data could be used for modeling consisted of extracted compound. The resulting normalized peak intensities formed a single matrix with Rt-*m*/*z* pairs for each file in the dataset. To reduce between-sample variation, we centered the imputed metabolic measures for each tissue sample on its median value and scaled it by its interquartile range (28). The *z*-score analysis scaled metabolites according to a reference distribution. The control samples were designated as the reference distribution. Thus, the mean and SD of the control samples were determined for each metabolite. Then, each sample was centered by the control mean and scaled by the control SD, per molecule. In this way, we can know how the metabolite expressions deviated from the control state. In addition, independent component analysis (ICA) was selected as the pattern recognition method (25).

### Western Blotting

RAW264.7 cells were lysed in 4 × loading buffer [250 mM Tris pH 6.8, 8% (w/v) SDS, 40% glycerol, 20% β-mercaptoethanol, and 0.01% bromophenol blue] and boiled for 10 min. After centrifugation, 50 μg of total protein extracts was separated by 12% SDS-PAGE, which was then transferred to nitrocellulose membranes for Western blotting. After blocking with 3% bovine serum albumin dissolved in Tris-buffered saline (TBS) containing 0.05% Tween-20 for 1 h at room temperature, the membranes were incubated with anti-phospho-Akt1-S473 or anti-β-actin primary antibodies at appropriate dilutions, followed by goat anti-rabbit or anti-mouse secondary antibodies conjugated with horseradish peroxidase, respectively. Positive band intensities were detected using a gel documentation system (LAS-3000 Fujifilm Medical Systems, Stamford, CT, USA).

### Therapeutic Effect of l-Valine or/and l-Arginine on Bacterial Eradication

Mice were acclimatized for 1 week and then randomly divided into groups for the investigation on the therapeutic effects of l-valine, l-arginine, or both. Mice were intraperitoneally challenged by bacterial pathogens (*K. pneumoniae*, MRSA, *P. aeruginosa*, or *E. coli* strains isolated from humans and chicken). Prior to killing by decapitation and extracting spleen, liver, and kidney tissues at 24 h, l-valine (0.5 g kg^−1^), l-arginine (0.25 g kg^−1^), l-valine (0.5 g kg^−1^) plus l-arginine (0.25 g kg^−1^) or an equal volume of sterile saline were intravenously administered to the bacteria-challenged mice at 1, 4, 7, 10, and 20 h through the tail vein. Replacement of l-valine or l-valine/l-arginine with d-valine or d-valine/l-arginine, respectively, was used as a control. The tissues were ground using sterile saline in gnotobasis. Plate counting was utilized to investigate bacterial eradication in the tissues. The homogenates were diluted in appropriate multiples and aliquots of the diluted homogenates were sampled in LB solid medium. Bacteria were counted when single colonies appeared in the medium after growing at 37°C. Differences between the groups were tested for significance at two significance levels (0.05 and 0.01) using the Statistical Package for the Social Sciences statistical software.

### Cell Culture and Quantitative Phagocytosis Assay

The murine macrophage cell line RAW264.7 was cultured at 37°C in a 5% CO_2_ incubator in DMEM (HyClone) supplemented with 10% (V/V) cosmic calf serum (HyClone), 100 U mL^−1^ penicillin G and 100 U/mL streptomycin. Macrophage phagocytosis was examined as described previously (26). Briefly, RAW264.7 cells were harvested using CaCl_2_- and MgCl_2_-free PBS containing 5 mM EDTA and plated at 5 × 10^6^ macrophages/well in 6-well plates. For experiments with administration of the indicated concentrations of l-valine, LPS, l-arginine, arginase inhibitor, or NO inhibitor, the cells were deprived of serum overnight and then incubated alone or additively with the abovementioned molecules for the indicated times in serum-starved media, including DMEM, l-valine-free medium (DMEM without l-valine), l-arginine-free medium (DMEM without l-arginine), and l-valine and l-arginine-free medium (DMEM without l-valine and l-arginine). After pretreating for 6 h, *E. coli*-GFP or FITC-conjugated *K. pneumoniae* cells were centrifuged onto macrophages at a multiplicity of infection of 100 in the indicated medium without serum or antibiotics. Then, the plates were placed either in 37°C or 4°C for the indicated times. After infection, the macrophages were vigorously washed with cold PBS to stop additional bacterial uptake or to destroy the bacteria in the phagosomes. Cells were washed at least four times in cold PBS and then fixed in 4% paraformaldehyde before being harvested in cold PBS containing 5 mM EDTA and subjected to FACS^®^ analysis.

### Ultra Performance Liquid Chromatography (UPLC)-MS Analysis for Extra- and Intracellular l-Valine and l-Arginine

l-valine or l-valine plus NO inhibitors were added into l-valine-free DMEM and then incubated with RAW264.7 cells. After 3 h incubation, 100 μL aliquots of medium were mixed with 400 μL of acetonitrile. The mixture was mixed by vortex for 2 min, followed by centrifugation at 14,000 rpm for 10 min at 4°C. The cells were lysed by sonication in 400 μL extraction solution (50% acetonitrile and double-distilled water). After centrifugation, the supernatants from medium or cell samples were transferred and diluted at a ratio of 1:1 with acetonitrile, which was used for subsequent analysis of UPLC/MS-MS. Approximately 90 μL ACN were added to 30 μl mice serum sample. The mixture was mixed by vortex for 1 min, followed by centrifugation at 12,000 rpm for 10 min at 4°C. Murine tissues were homogenized in a laboratory homogenizer for 3 min. Then, 1 mL ACN was added to the sample. The samples were disrupted by sonication for 2 min, followed by centrifugation at 12,000 rpm for 10 min at 4°C. The supernatant was collected and analyzed by UPLC-MS/MS.

Ultra performance liquid chromatography analysis was performed on a Waters ACQUITY UPLC system equipped with an Acquity BEH C_18_ column (50 mm × 2.1 mm i.d., 1.7 μm; Waters Corp.). The sample was injected during the loading step by the loading pump and auto-sampler onto the column. Separation was using linear gradient elution with mobile phase A (acetonitrile) and B (10 mM ammonium acetate with 0.1% formic acid in ultra-pure water) at a flow rate of 0.25 mL min^−1^. The gradient elution was as follows: 0–0.5 min, 95% A; 0.5–2 min, 20% A; 2–2.5 min, 20% A; 2.5–4 min, 95% A. The injection volume was 10 μl, and the column temperature was kept at 35°C. Mass spectrometry detection was carried out in QUATTRO PREMIER XE equipped with an electrospray ionization source operating in positive ionization mode (ESI+). The capillary voltage was set to 3,000 V; the cone voltage was set to 20 V. The extractor voltage and RF Lens were set at 1 and 0.5 V, respectively. The desolvation gas flow was set to 650 L h^−1^ at temperature of 450°C, the cone gas flow rate was set at 50 L h^−1^, and the source temperature was set at 120°C. Identification was measured in MRM mode, the precursor ion and quantifier ion of l-arginine and l-valine was 175 > 70 and 118 > 72.

### NO, Urea Concentration, and Arginase Activity Measurements

Total NO concentration in culture medium and cells was calculated by measuring the nitrate and nitrite concentrations with a Total Nitric Oxide Assay Kit (Beyotime, China) according to the manufacturer’s instructions. The optical densities at 540 nm were recorded using a Microplate Reader (Thermo MutliscanMK3; Thermo Fisher Scientific, Waltham, MA, USA). The concentration of NO output was calculated from the standard curve. Urea production was determined using a Urea Colorimetric Assay Kit (BioVision). Five million macrophages were harvested and lysed for 30 min in 100 μL of 10 mM Tris–HCl, pH 7.4, containing 0.4% (w/v) Triton X-100. Then, the cells were centrifuged at 13,000 rpm for 10 min. The supernatant was collected for the Arginase Activity Assay kit (Sigma, MAK112). The quantitative data were expressed as the mean ± SD. One-way ANOVA was used to determine the statistical significance.

### Effect of Mouse Serum on Killing *K. pneumoniae* in the Absence and Presence of l-Valine

l-valine (0.5 g kg^−1^) or equal volumes of sterile saline were intravenously administered to the mice through the tail vein. Two hours later, serum was collected from these mice. *K. pneumoniae* at an absorbance of 1.0 (OD600) in LB medium were harvested by centrifugation at 7,000 rpm for 15 min at 4°C. The cultures were washed and then resuspended in 0.85% NaCl. Equal amounts of *K. pneumoniae* (~10^4^ CFU) were added into the serum of both groups in a final volume of 150 μL. Samples were incubated at 37°C for 24 h with slow rotation. *K. pneumoniae* dilutions were plated on LB agar for colony formation.

### Effect of Valine and Arginine on Bacterial Growth

*Klebsiella pneumoniae*-0367 and *E. coli* Y17 were cultured in LB medium for 16 h at 37°C. Samples were harvested at 7,000 rpm for 5 min, washed three times with 30 mL sterile saline and resuspended in sterile saline to 0.6 at OD600. Different concentrations of valine or arginine were added to the samples to reach a final volume of 5 mL; no metabolite was used a control. Samples were incubated at 37°C for 6 h. After incubation, 100 μL aliquot samples were removed periodically, serially diluted, and plated on LB agar. The plates were incubated at 37°C for 8–10 h. Only those dilutions that generated 20–200 clones were counted to calculate the CFUs. Percent survival was determined by dividing the CFU obtained from a treated sample by the CFU obtained for the control.

## Results

### Metabolomic Profiling of Plasma from Surviving and Dead Mice following *K. pneumoniae* Infection

**Figure 1:**
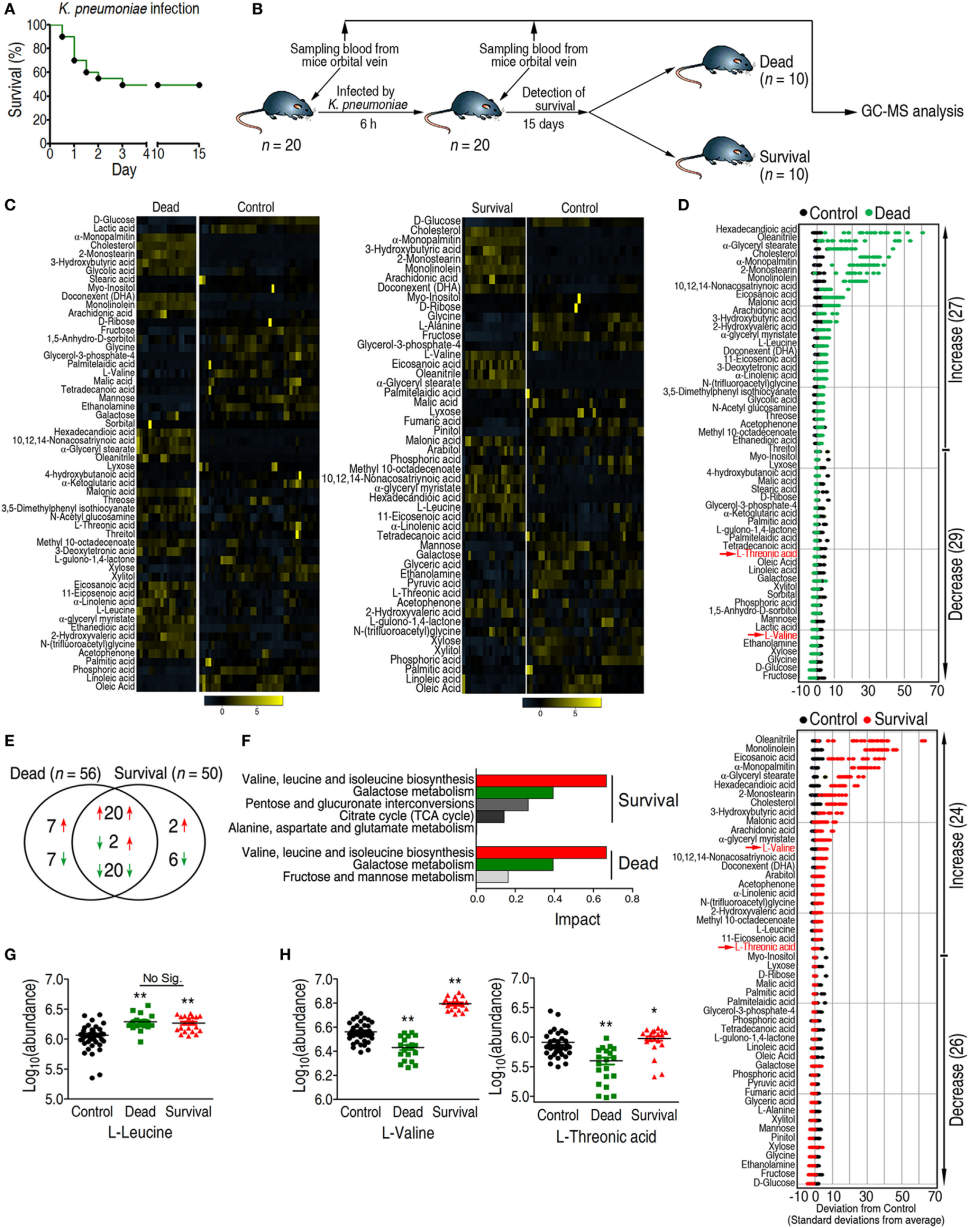
**Differential analysis coupled with pathway enrichment analysis identifies l-valine as a potential anti-infective metabolite**. **(A)** Survival percentage for mice infected with sublethal doses of *Klebsiella pneumoniae*. Based on the pretest, the sublethal dose of *K. pneumoniae* was determined to be 1 × 10^8^ CFU. **(B)** Experimental design for sample acquisition for gas chromatography–mass spectrometry (GC-MS)-based metabolomics. Prior to infection, 100 μL blood was drawn from the orbital vein in each of 20 mice as the control group. Eighteen hours later, a half-lethal dose of *K. pneumonia* was intraperitoneally injected into each mouse. Six hours after infection, 100 μL blood was collected. Survival of all the mice was observed for 15 days. **(C)** Heat map showing the relative abundance of the 56 and 50 significantly differential metabolites in the dead and survival groups as indicated, respectively. **(D)** *Z*-score plots corresponding to the data in panel **(C)**. The upper panel is the dead group, and the lower panel is the survival group. **(E)** Venn diagram showing the overlap of differential metabolites between the dead and survival groups. Decreased and increased metabolites are indicated with green and red arrows, respectively. **(F)** Pathway enrichment of differential metabolites in the dead and survival groups. A horizontal histogram was selected to show the impact of the enriched pathway with impact values >0.1. **(G,H)** Abundance of l-leucine, l-valine, and l-threonic acid in the control, dead, and survival groups. Error bars ± SEM, *p < 0.05 and **p < 0.01.

Mice infected with LD_50_ *K. pneumoniae* (No. 0367) producing TEM-type ESBLs (Figure S1 in Supplementary Material) displayed two consequences: either succumbing to or surviving the infection (Figure 1A). Plasma samples were drawn from the mice 6 h post-infection; serum drawn before infection served as the control group (Figure 1B). Metabolic profiling of the plasma samples was performed using a GC-MS-based approach followed by multivariate analysis to identify crucial biomarkers. The reliability of GC-MS was assessed through correlation coefficients with two technical repeats (Figure S2A in Supplementary Material). A total of 68 metabolites were detected in each sample; internal standard and solvent peaks were excluded. The metabolites are displayed as a heat map and *Z*-score plot (Figures S2B,C in Supplementary Material). ICA identified two independent components, IC01 and IC02, whereby IC01 differentiated three groups without any significant outliers (Figure S2D in Supplementary Material), indicating the reproducibility of the samples.

### Pattern Recognition Identifies l-Valine As a Potential Anti-infection Metabolite

A two-sided Wilcoxon rank-sum test coupled with a permutation test was used to identify crucial biomarkers that differentiated these three groups (27). Compared with the control group, the abundance of 56 and 50 metabolites were significantly altered in the dead and survival groups (*p* < 0.05), respectively (Figures 1C,D), among which 42 metabolites were shared. Among the 42 metabolites, 20 metabolites were increased, 20 metabolites were decreased, and 2 metabolites were increased in the survival group but lower in the dead group. In addition to the shared metabolites, seven metabolites were increased, and seven metabolites were decreased in the dead group, whereas two metabolites were increased and six metabolites were decreased in survival group (Figure 1E).

In the pathway analysis, the differential abundance of 50 metabolites in the survival group and 56 metabolites in the dead group enriched for four and three pathways (*p* < 0.05 and impact > 0.1), respectively (Figure 1F). The shared pathways were valine, leucine, and isoleucine metabolism, and galactose metabolism. All of the detected metabolites from galactose metabolism, including d-glucose, mannose, fructose, galactose, and myo-inositol, were decreased in both the survival and dead groups (Figure S2E in Supplementary Material). Two metabolites, l-valine and l-leucine, were enriched in valine, leucine, and isoleucine metabolism. Although the abundance of l-leucine was increased in both the survival and dead groups, no significant differences were detected between the two groups (Figure 1G). By contrast, l-valine and l-threonic acid were differentially expressed between the two groups (Figures 1D,H). The abundance of l-valine was higher than l-threonic acid in the survival group. Therefore, l-valine could be a prognostic biomarker for *K. pneumoniae* infection and could act as a modulator for protecting the host against infection.

### Exogenous l-Valine Displays an Anti-infective Effect on Bacterial Infection

**Figure 2:**
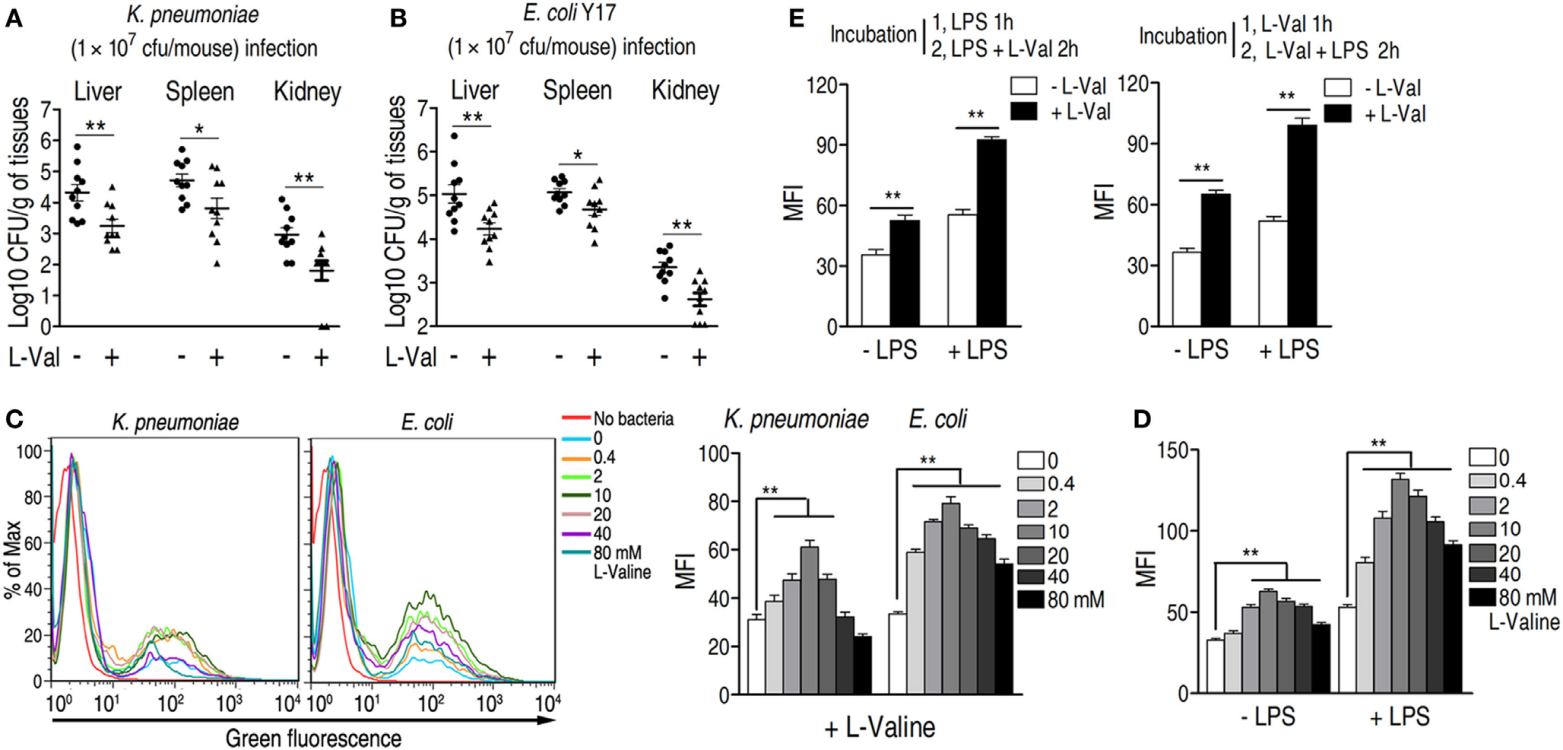
**Effect of exogenous l-valine on bacterial load in infected mice and macrophage-mediated phagocytosis**. **(A,B)** l-valine promotes the clearance of clinical **(A)** *Klebsiella pneumoniae* (No. 0367) and **(B)** *Escherichia coli* Y17 in mice (*n* = 10). **(C)** Representative flow charts (left) and the mean fluorescence intensity (MFI) (MFI, right) of FITC-conjugated *K. pneumoniae* and GFP-*E. coli* in l-valine-pretreated RAW264.7 cell at the indicated concentrations at 3 h. **(D)** Effect of exogenous l-valine on the phagocytosis of GFP-*E. coli* in the absence and presence of lipopolysaccharide (LPS) administration. **(E)** Synergetic effects of LPS and l-valine on macrophage phagocytosis. The left histogram indicates the MFI of GFP-*E. coli* in macrophages treated with LPS for 1 h and subsequently treated with LPS plus l-valine for 2 h. The right histogram reveals the MFI of GFP-*E. coli* in macrophages treated with l-valine for 1 h and subsequently treated with LPS plus l-valine for 2 h. Error bars ± SEM, *p < 0.05 and **p < 0.01.

The concentrations of l-valine in the control, dead, and survival mice were 9, 5, and 17 μM, respectively, as normalized to the internal standard control ribitol (0.1 mg mL^−1^, 5 μL). Thus, l-valine levels should increase at least two-fold to promote survival during bacterial infection. To examine the potential anti-infective role of l-valine *in vivo*, two groups of mice were i.p. injected with *K. pneumoniae*, followed by i.v. injection with l-valine (0.5 g kg^−1^) or sterile saline five times within 20 h. The levels of l-valine, but not l-arginine, were significantly elevated in plasma at different time points (Figures S3A,B in Supplementary Material). All of the mice were sacrificed at 24 h after infection. The liver, spleen, and kidney were removed surgically for analysis of bacterial load and l-valine. Bacterial counts were significantly lower and l-valine was significantly higher in the liver, spleen, and kidney of the mice injected with l-valine than those in the saline control group (Figure 2A; Figure S3C in Supplementary Material). Similar results were obtained when the mice were exposed to a clinical strain of antibiotic-resistant *E. coli* Y17 (Figure 2B). The replacement of l-valine with d-valine had a similar bacterial load as the saline control (Figures S4A,B in Supplementary Material), thus excluding the non-specific effect of l-valine on mice. These data strongly suggest that l-valine decreases bacterial load in *K. pneumonia-* and *E. coli-*infected mice.

To exclude the possibility that l-valine exerts its effects through the complement system or immunoglobulins, we investigated the effect of l-valine on bacterial growth in the presence of freshly drawn murine serum. The growth of bacteria was unaffected even in the presence of l-valine (Figure S4C in Supplementary Material), implying that l-valine-mediated bacterial elimination was unlikely attributed to the complement system and immunoglobulins. Thus, to further explain the anti-infective effects of l-valine on infected mice, we surmised that l-valine might stimulate phagocytosis, which would thereby increase the rate at which pathogens were eliminated from the host. This hypothesis was tested *in vitro* by examining the cytoplasmic mean fluorescence intensity (MFI) in murine macrophages (RAW264.7). l-Valine-pretreated RAW264.7 cells were incubated with FITC-conjugated *K. pneumoniae* or green fluorescent protein (GFP)-expressing *E. coli*. l-valine markedly stimulated the phagocytosis of fluorescence-tagged bacterial pathogens at 0.4–10 mM in a dose-dependent manner (Figure 2C). However, phagocytosis was not changed in d-valine -pretreated RAW264.7 cells (Figure S4D in Supplementary Material). Other amino acids, such as glycine, were unable to enhance phagocytosis (Figure S4E in Supplementary Material). These results support the immunomodulatory function of l-valine in macrophages. Notably, high doses of l-valine (20–40 mM) had no significant impacts on normal phagocytosis levels, even though high l-valine doses had fewer effects than lower l-valine doses (Figure 2C). Additionally, l-valine alone did not affect bacterial growth (Figure S4G in Supplementary Material). Plate counting revealed that incubation with l-valine reduced the bacterial load in extracellular environments and enhanced the bacterial load within cells (Figure S4H in Supplementary Material).

During infection with Gram-negative bacteria, including *K. pneumoniae* and *E. coli*, LPS is abundantly produced and therefore contributes to immune response. Therefore, the effect of l-valine on phagocytosis was examined upon LPS stimulation. Macrophages treated with l-valine displayed higher phagocytosis than untreated macrophages, even in the presence of LPS (Figure 2D). Additionally, phagocytosis was also further boosted by pretreatment with LPS for 1 h as well as when followed by 2 h LPS plus l-valine treatment or by 1 h l-valine followed by 2 h l-valine plus LPS treatment (Figure 2E). These data support that l-valine enhances macrophage-mediated innate immunity to Gram-negative pathogens.

### l-Valine-Induced PI3K/Akt1 Activation and NO Production Contribute to the Enhanced Phagocytosis

**Figure 3:**
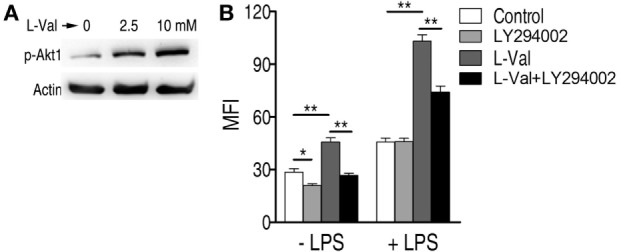
**Activation of PI3K/Akt by exogenous l-valine is essential for increased phagocytosis**. **(A)** Phosphorylation of Akt1 at Ser473 was examined in RAW264.7 cells treated with l-valine for 3 h. **(B)** Mean fluorescence intensity (MFI) of GFP-*E. coli* in RAW264.7 cells exposed to l-valine in the absence or presence of LY294002 (10 μM), an inhibitor of PI3K. Error bars ± SEM, *p < 0.05 and **p < 0.01.

A previous study showed that depletion of extracellular l-valine in DCs decreased the mTORC1/S6K-signaling pathway (16), which is activated by PI3K/Akt (33). Consistently, l-valine-treated macrophages displayed increased phospho-Akt1 (p-Akt1) (Figure 3A). When macrophages were treated with the PI3K inhibitor LY294002 (10 μM), the macrophage MFI was lower than in untreated cells, regardless of the presence of LPS (Figure 3B). In particular, upon LPS stimulation, LY294002 obviously reduced almost half of the phagocytosis enhanced by l-valine (Figure 3B). Thus, l-valine-induced activation of PI3K/Akt1 is partly responsible for the improved phagocytosis.

**Figure 4:**
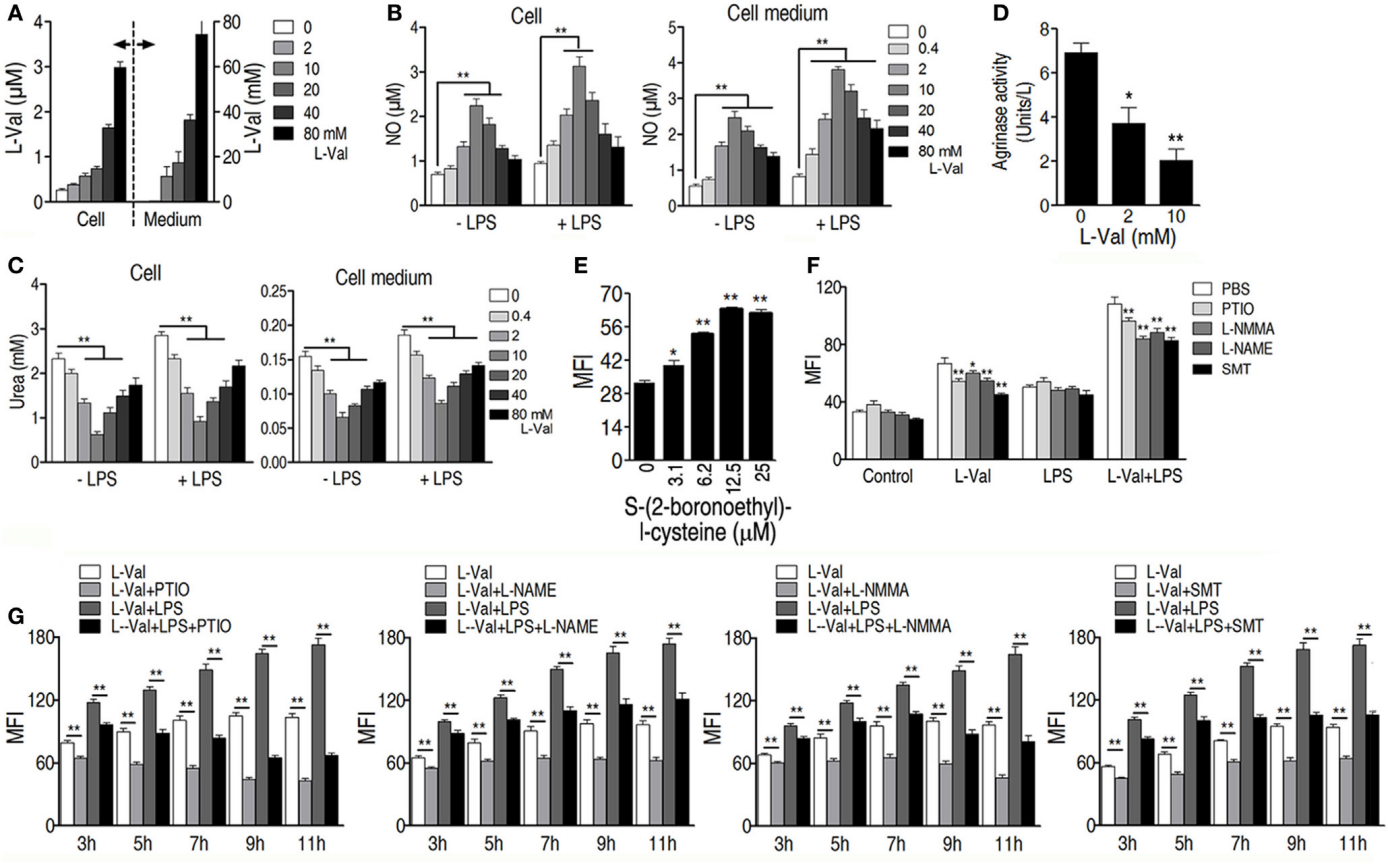
**Exogenous l-valine enhances macrophage phagocytosis by boosting NO production**. **(A)** Detection of extra- and intracellular l-valine in l-valine-stimulated macrophages. **(B)** Effect of exogenous l-valine on NO production by macrophages in the absence or presence of lipopolysaccharide (LPS). **(C)** Effect of l-valine on urea production by macrophages in the absence or presence of LPS. **(D)** l-valine inhibited arginase activity in RAW264.7 cells. **(E)** The potent and specific arginase inhibitor *S*-(2-boronoethyl)-l-cysteine suppressed macrophage phagocytosis in a dose-dependent manner. **(F)** Effect of NO inhibitors on l-valine-stimulated phagocytosis by macrophages in the absence or presence of LPS at 3 h. **(G)** Effect of NO inhibitors on l-valine-stimulated phagocytosis by macrophages at the indicated times and in the absence or presence of LPS. Error bars ± SEM, *p < 0.05 and **p < 0.01.

It has also been reported that the addition of l-valine led to a concentration-dependent decrease in urea and NO production in tissues and endothelial cells when arginase is inhibited (34, 35). We therefore hypothesized that NO production was involved in l-valine-enhanced phagocytosis. First, exogenous l-valine increased intracellular levels of l-valine as quantified by UPLC-MS (Figure 4A), indicating that l-valine functions inside macrophages. Consistent with a previous finding (34), exogenous l-valine concentrations ranging from 0.4 to 10 mM prompted NO production but reduced urea production extra- and intracellular levels in a dose-dependent manner. Higher concentrations of l-valine had weaker effects (Figures 4B,C). LPS stimulation further enhanced l-valine-induced NO production (Figure 4B). Meanwhile, l-valine inhibited RAW264.7 cell arginase activity (Figure 4D). The inhibition of arginase activity with *S*-(2-boronoethyl)-l-cysteine, a potent and specific inhibitor, significantly boosted macrophage phagocytosis (Figure 4E). These data provide a strong interrelationship between l-valine-induced phagocytosis and NO production. To further prove this idea, we investigated the effects of four other NO inhibitors, carboxy-PTIO (PTIO, NO scavenger), l-NMMA (inhibition of total NO synthase), SMT [inhibition of inducible NO synthase (iNOS)], and l-NAME (inhibition of endothelial NO synthase), on l-valine-induced phagocytosis. As expected, these inhibitors significantly suppressed l-valine-induced macrophage phagocytosis with or without LPS treatment (Figure 4F), and MFI decreased in a time-dependent manner (Figure 4G). Together, these data reveal that PI3K/Akt1 activation and NO production all contribute to l-valine-enhanced phagocytosis.

### l-arginine Is Involved in l-Valine-Mediated Phagocytosis

**Figure 5:**
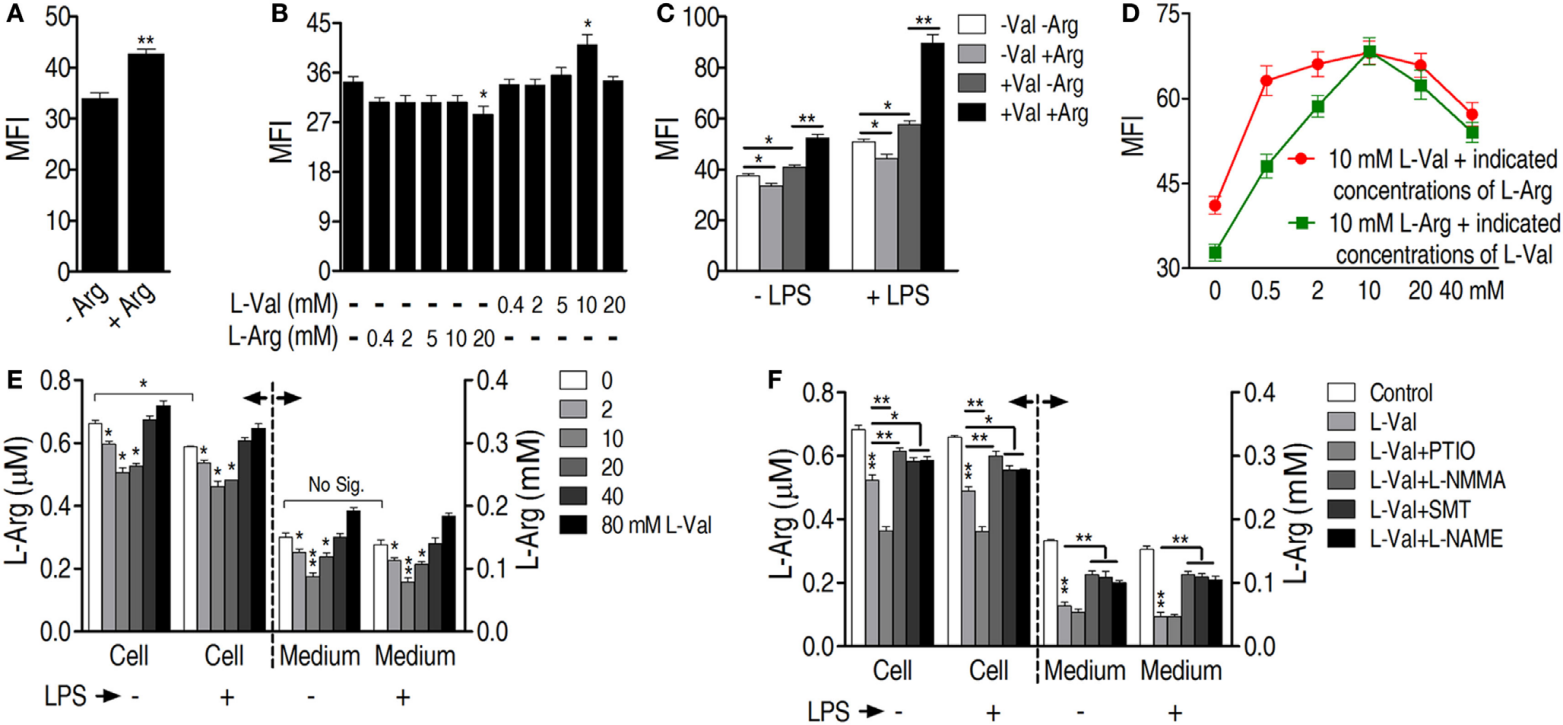
**l-Arginine is required for l-valine-mediated phagocytosis**. **(A)** l-Arginine (0.4 mM) is required for basal macrophage phagocytosis. RAW264.7 cells are cultured in two types of DMEM, l-arginine-free DMEM and DMEM containing 0.4 mM l-arginine. Phagocytosis of GFP-*E. coli* was quantified by flow cytometry [macrophage mean fluorescence intensity (MFI)]. **(B)** By employing DMEM deficient in both l-valine and l-arginine as the background medium, exogenous l-valine only promotes a slight increase in phagocytosis of GFP-*E. coli*, whereas exogenous l-arginine is not effective and even counters phagocytosis. Different concentrations of l-valine or l-arginine are added to DMEM deficient in both l-valine- and l-arginine, and then GFP-*E. coli* phagocytosis is measured by flow cytometry. **(C)** l-arginine promotes l-valine-mediated phagocytosis in the absence or presence of lipopolysaccharide (LPS). DMEM without both l-valine- and l-arginine is used as background medium and 10 mM l-valine, 0.4 mM l-arginine, and 10 mM l-valine plus 0.4 mM l-arginine are added. Phagocytosis of GFP-*E. coli* is investigated in the absence or presence of LPS by flow cytometry. **(D)** Different combinations of l-valine and l-arginine have varying effects on the phagocytosis of GFP-*E. coli*. The results shown by a red line indicate that macrophage phagocytosis is synergistic with 10 mM l-valine at different l-arginine concentrations. The results shown by a green line reveal that macrophage phagocytosis is synergistic with 10 mM l-arginine and different l-valine concentrations. The background medium is DMEM without both l-valine and l-arginine. **(E)** l-arginine levels are measured in l-valine-incubated macrophages in the absence or presence of LPS by using ultra performance liquid chromatography (UPLC)-MS. **(F)** NO inhibitors eliminated the effects of l-valine on l-arginine levels in the absence or presence of LPS. The l-arginine levels in the cells and medium are detected by UPLC-MS analysis. Error bars ± SEM, *p < 0.05 and **p < 0.01.

Because l-arginine is the exclusive source of NO in cellular metabolism, the role of l-arginine in l-valine-mediated phagocytosis was investigated. Removal of l-arginine from the culture medium reduced basal phagocytosis levels (Figure 5A). l-arginine does not affect bacterial survival (Figure S4D in Supplementary Material). However, replacement of l-arginine with d-arginine had no effect on macrophage phagocytosis (Figure S4G in Supplementary Material), indicating the specific effect of l-arginine on phagocytosis. When different concentrations of l-arginine or l-valine were added to medium deficient in both l-valine and l-arginine, respectively, exogenous l-arginine was not effective and even countered phagocytosis (Figures 5B,C). However, the addition of 10 mM l-valine increased phagocytosis in the absence or presence of LPS (Figures 5B,C). Therefore, we believed that the increased phagocytosis that occurred at 10 mM in valine-incubated cells was specific and might be caused by eNOS, which is constitutively expressed in RAW264.7 mouse macrophages (36). We proposed that 10 mM valine was able to inhibit arginase activity, thereby elevating NO production through eNOS metabolism, which thus enhanced phagocytosis. However, the MFI increase was very slight because of the limited l-arginine within the cells (Figure 5B). Lower concentrations of valine probably did not optimize the inhibition of arginase activity and higher concentrations, such as 40 mM valine, might promote cellular side effects (see details in Section “Discussion”), thereby resulting in the decrease in the MFI. Macrophages displayed the highest levels of phagocytosis when 10 mM l-valine and l-arginine were used (Figure 5D). Regardless of the presence of LPS, l-arginine was decreased in l-valine-treated macrophages and macrophage-cultured medium (Figure 5E), which could be restored by additional treatment with NO inhibitors, except for PTIO (Figure 5F). It is possible that PTIO is a specific NO scavenger and has limited effects on NO synthase activity, which directly metabolizes l-arginine to produce NO, thereby maintaining or even increasing the consumption rate of l-arginine. Together, these data indicate that l-arginine is essential for the l-valine-induced phagocytosis and potentially has a synergetic effect on bacterial elimination *in vivo*.

### l-Valine and l-Arginine Synergistically Protect Mice against Clinically Relevant Multidrug-Resistant Bacteria

**Figure 6:**
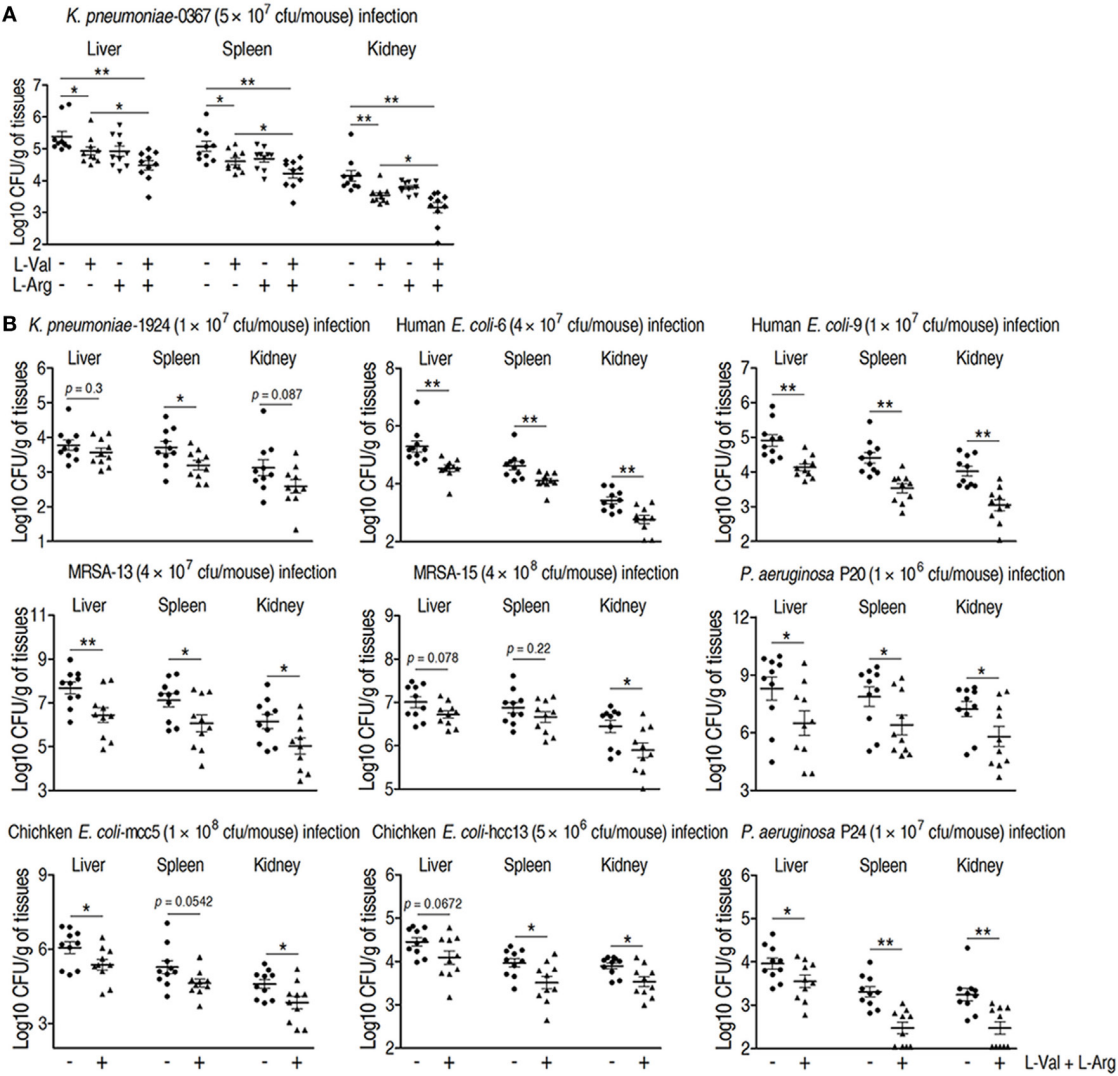
**Therapeutic effects of l-valine and l-arginine synergistic administration on mice infected with clinically relevant multidrug-resistant bacteria**. **(A)** Mice administered combined l-valine with l-arginine display stronger elimination of clinical *Klebsiella pneumoniae* than mice are administered l-valine or l-arginine alone (*n* = 10). **(B)** Administration of l-valine with l-arginine likely protects mice against other clinical isolates including *K. pneumoniae*, methicillin-resistant *Staphylococcus aureus* (MRSA), *Pseudomonas aeruginosa*, human-derived *Escherichia coli*, and chicken-derived *E. coli*. Error bars ± SEM, *p < 0.05 and **p < 0.01.

Clinically relevant multidrug-resistant bacteria are associated with therapy failures and public health crises that are hard to be or even no longer controlled by antibiotics, which must be addressed with new treatments (37, 38). Therefore, it would be clinically helpful if the combined administration of l-valine and l-arginine promotes innate immunity-dependent killing of these multidrug-resistant bacteria. To test this idea, *K. pneumonia*-challenged mice were treated with l-valine, l-arginine, or both via intravenous injection. l-valine alone enhanced the elimination of *K. pneumoniae* by the host, whereas injection of both of l-valine and l-arginine had stronger effects (Figure 6A), which was supported by elevated l-valine and l-arginine levels in the plasma and kidney (Figures S3A,B,D in Supplementary Material). However, neither d-valine nor d-arginine alone could eliminate *K. pneumoniae* and *E. coli* Y17 (Figures S4A,B in Supplementary Material), indicating the specific effects of l-valine and l-arginine. Valine–arginine combination therapy was also tested in other clinical multidrug-resistant pathogens, including Gram-positive and -negative bacteria isolated from humans or chickens; this combination also displayed therapeutic effects against these pathogens (Figure 6B). These findings indicate that valine–arginine combination therapy could be a useful intervention in infectious diseases caused by multidrug-resistant bacteria.

## Discussion

**Figure 7:**
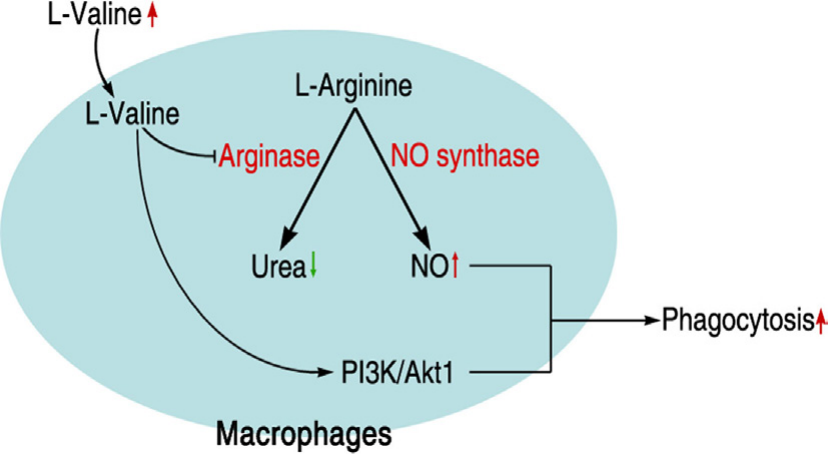
**Schematic model for l-valine-induced phagocytosis in macrophages**.

Intensive and inappropriate use of antibiotics dramatically leads to the development of drug resistance in bacterial pathogens, increasing the risk of severe diseases or death after exposure to multidrug-resistant bacteria (5, 39). Although antibiotics are still the first choice for treating such infections, severe consequences would be expected if more multidrug-resistant bacteria were generated and spread. A novel strategy for controlling such a situation is urgently needed. Our previous studies strongly suggested that the harnessing alanine and glucose, metabolites that are suppressed in antibiotic-resistant bacteria, could revert such a phenotype (24). The idea of using metabolites to reprogram existing metabolic pathways to a way that we want has been tested in many other species (23). Here, we show that metabolite-mediated reprogramming is not limited to bacteria, but is possible in the host as well. We identified that l-valine is a key metabolite for promoting mouse survival under *K*. *pneumoniae* challenge; this effect could also be observed with other pathogens such as *E. coli, P. aeruginosa*, or MRSA, implying that l-valine may be a metabolite that regulates immune functions. Not surprisingly, we found that l-valine actually increases macrophage phagocytosis in an l-arginine-dependent manner. The enhanced phagocytosis was attributed to increased PI3K/Akt1 activation and NO production (Figure 7). Thus, our study not only proposes the use of metabolites in managing bacterial infection but also presents a well-established platform for identifying metabolites for bio-reprogramming.

l-valine, an essential amino acid, has more functions than just in nutrition. In a previous study, mice fed with synthetic diets with limited l-valine exhibited obviously increased susceptibility to bacterial infection (40), indicating the anti-infective potential of l-valine; valine supplementation is not currently used in the clinic. However, the mechanism underlying this anti-infection property is unknown. A recent paper revealed the immunological function of l-valine in DCs and demonstrated that l-valine deficiency inhibits the differentiation of monocytes into mature DCs as well as IL-12 production, likely by downregulating the mTORC1/S6K signaling pathway, which may be the cause of enhanced sensitivity to bacterial infection in cirrhotic patients (16). Normally, PI3K/Akt is the upstream activator of mTORC1 (33, 41). Our study found that l-valine is capable of boosting PI3K/Akt1 activation in macrophages. PI3K inhibition partially reduced the l-valine-induced phagocytosis of bacteria, which is consistent with previous studies showing that PI3K/Akt activation mediates macrophage phagocytosis (42, 43).

We also addressed whether the mechanism of l-valine-enhanced phagocytosis of bacteria in macrophage is associated with NO production. Although phagocytosis and NO production are believed to clear pathogens, their intercorrelation has not been actually established. Our study revealed that l-valine induced macrophages to engulf bacteria through NO and that NO played a major role in the direct intracellular-mediated mechanism that leads to killing pathogens (44). Meanwhile, NO produced by iNOS functions as a signaling molecule, which could strengthen the phagocytosis by LPS-stimulated or IFN-γ-primed macrophages (45, 46). Furthermore, engagement of Fcγ-receptors triggered neuronal and endothelial NOS activity (nNOS and eNOS), both of which produced low levels of NO that functioned to promote macrophage phagocytosis (47). The present study showed that iNOS and eNOS were the two major metabolic enzymes that produce NO in l-valine-treated macrophages. Inhibitors of iNOS and eNOS reduced l-valine-enhanced phagocytosis. In fact, promoting NO production with l-valine has been documented in the literature (34, 35). However, the ability of l-valine to enhance NO production and in turn increase bacterial phagocytosis by macrophages was previously unrecognized. However, it is helpful if primary murine macrophages, NOS KO macrophages, and human macrophages can be used to further confirm our conclusions.

Another finding in this study is that l-arginine was essential for l-valine-enhanced phagocytosis and NO production. l-arginine is the sole metabolic source for NO production. Factors that limited the availability of l-arginine could reduce the production of NO, thereby increasing host susceptibility to invading pathogens (48). In mammalian cells, arginase and NOS compete with one another for l-arginine as an enzyme substrate. Deprivation of l-arginine leads to the reduced translational efficiency of iNOS mRNA and stability of iNOS protein (49, 50). Although the enzymatic activities of arginase and NOS are co-induced in macrophages in response to bacterial infection, the role of arginase is obviously stronger than that of NOS (51, 52), indicating that arginase is the predominant regulator of arginine availability in activated macrophages. Furthermore, inhibition of arginase activity in macrophages increases host survival during *Toxoplasma gondii* infection and reduces the bacterial burden in the lung during tuberculosis infection (52). These data are consistent with our finding that arginase increases the survival of clinically relevant multidrug-resistant isolates. Addition of l-arginine reinforces that phagocytosis in l-valine-treated macrophages and valine–arginine combination therapy further reduces the bacterial load in tissues, proving the significance of l-arginine availability in eliminating the bacterial infection.

Although the findings on exogenous metabolite-induced cellular l-valine/arginine elevation and l-valine-enhanced phagocytosis *in vitro* correlate with elevated host l-valine/arginine and bacterial control *in vivo*, further investigation is required to determine whether the *in vitro* mechanism is relevant to bacterial control *in vivo*, which includes examining whether the macrophages are really targeted *in vivo* by l-valine and l-arginine administration and determining whether other immune cells are involved (53). Additionally, two phenotypes of unknown cause from the present study will also need to be determined in future studies. One is how increasing concentrations of l-valine entail weaker effects on urea/NO synthesis (Figures 4B,C). In mammals, degradation of l-valine generates propionyl-CoA, which can be metabolized to succinyl-CoA by sequential catalytic reactions, including propionyl-CoA carboxylase and methylmalonyl-CoA racemase. Succinyl-CoA feeds into the Krebs cycle and produces NADH, which results in ATP production through mitochondrial respiration. During this respiration, reactive oxygen species (ROS), a pathway byproduct, are generated due to electron transfer to O_2_ (54). Low concentrations of mitochondrial ROS augment macrophage bactericidal activity (55); however, high concentrations of mitochondrial ROS may pose a barrier for macrophage survival (54, 56). Therefore, we propose that increasing concentrations of l-valine induces overloaded mitochondrial ROS, which leads to macrophage dysfunction, thereby weakening the activity of NOS and eventually promoting weaker effects on urea/NO synthesis. Another phenotype is that phagocytosis is inhibited by 20 mM l-arginine supplementation in the absence of l-valine (Figure 5B). As mentioned above, the products of arginase catalysis are urea and l-ornithine, of which l-ornithine may be used for the synthesis of polyamines by macrophages (57). High concentrations of polyamines have the ability to inhibit NOS activity (58, 59). After l-valine deprivation, excess l-arginine is metabolized by arginase to produce abundant polyamines, the latter of which potentially suppress the activity of eNOS, a constitutively expressed enzyme in RAW264.7 cells (36). This process thereby reduces eNOS-mediated NO production and eventually decreases NO-mediated phagocytosis by macrophages.

In summary, we demonstrated that mouse resistance to bacterial infection is strongly associated with metabolic states. High levels of l-valine are vital for survival strategies by pathogen-infected hosts. Although how the host accumulates high levels of l-valine *in vivo* upon bacterial infection is unknown, our study provides a new clue to identifying metabolic modulators and highlights the possibility of employing this metabolic modulator-mediated innate immunity as a therapy for bacterial infections.

## Author Contributions

BP, X-hC, and X-xP wrote the manuscript. X-xP, T-cZ, and BP conceptualized and designed the project. X-xP, BP, HL, and X-hC interpreted the data. X-hC, BP, J-xZ, and HL performed data analysis. X-hC performed experiments. All the authors reviewed the manuscript.

## Conflict of Interest Statement

The authors declare that the research was conducted in the absence of any commercial or financial relationships that could be construed as a potential conflict of interest.
