# Supplementary material for: Exogenous l-Valine Promotes Phagocytosis to Kill Multidrug-Resistant Bacterial Pathogens
Source: Front Immunol. 2017 Mar 6;8:207. doi: 10.3389/fimmu.2017.00207 (PMC5337526; doi:10.3389/fimmu.2017.00207)
Supplement: Supplementary file 1 [file Data_Sheet_1.DOCX]

**
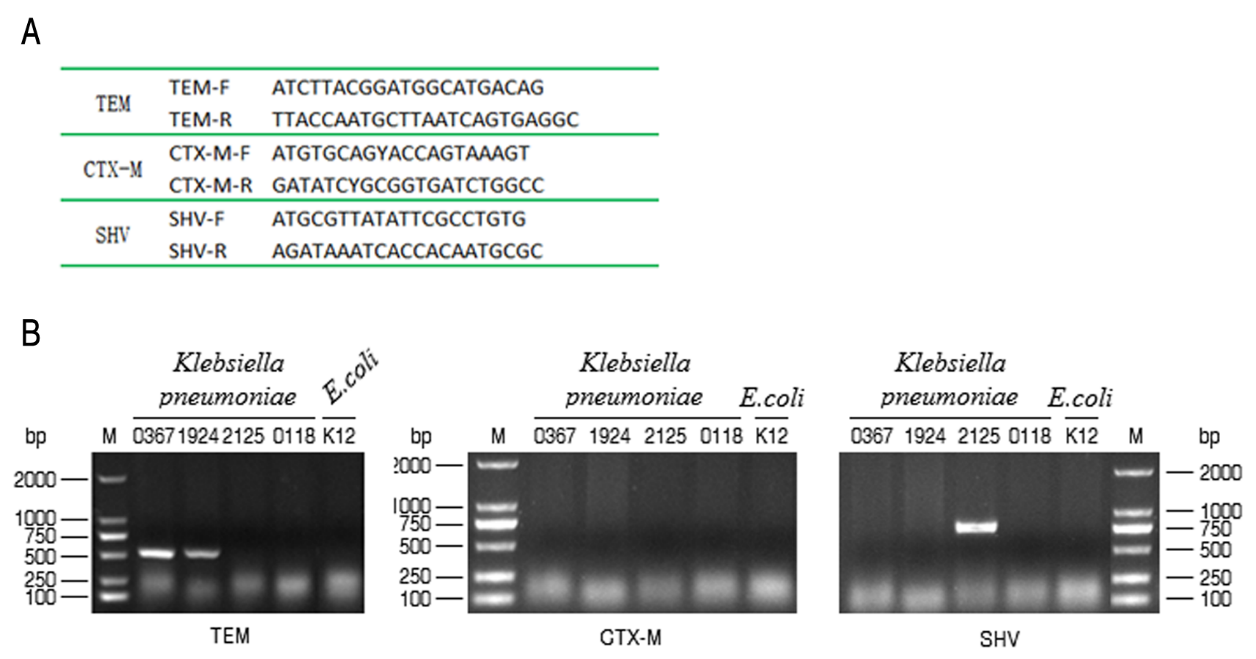
**

**Supplementary Figure 1. Confirmation of ESBL genotype of *K. pneumoniae* by polymerase chain reaction (PCR). (A)** Primers used in characterizing ESBL genotypes. **(B)** Identification of ESBLs in *K. pneumoniae* isolates by PCR. *E. coli* K12 was used as a negative control. **
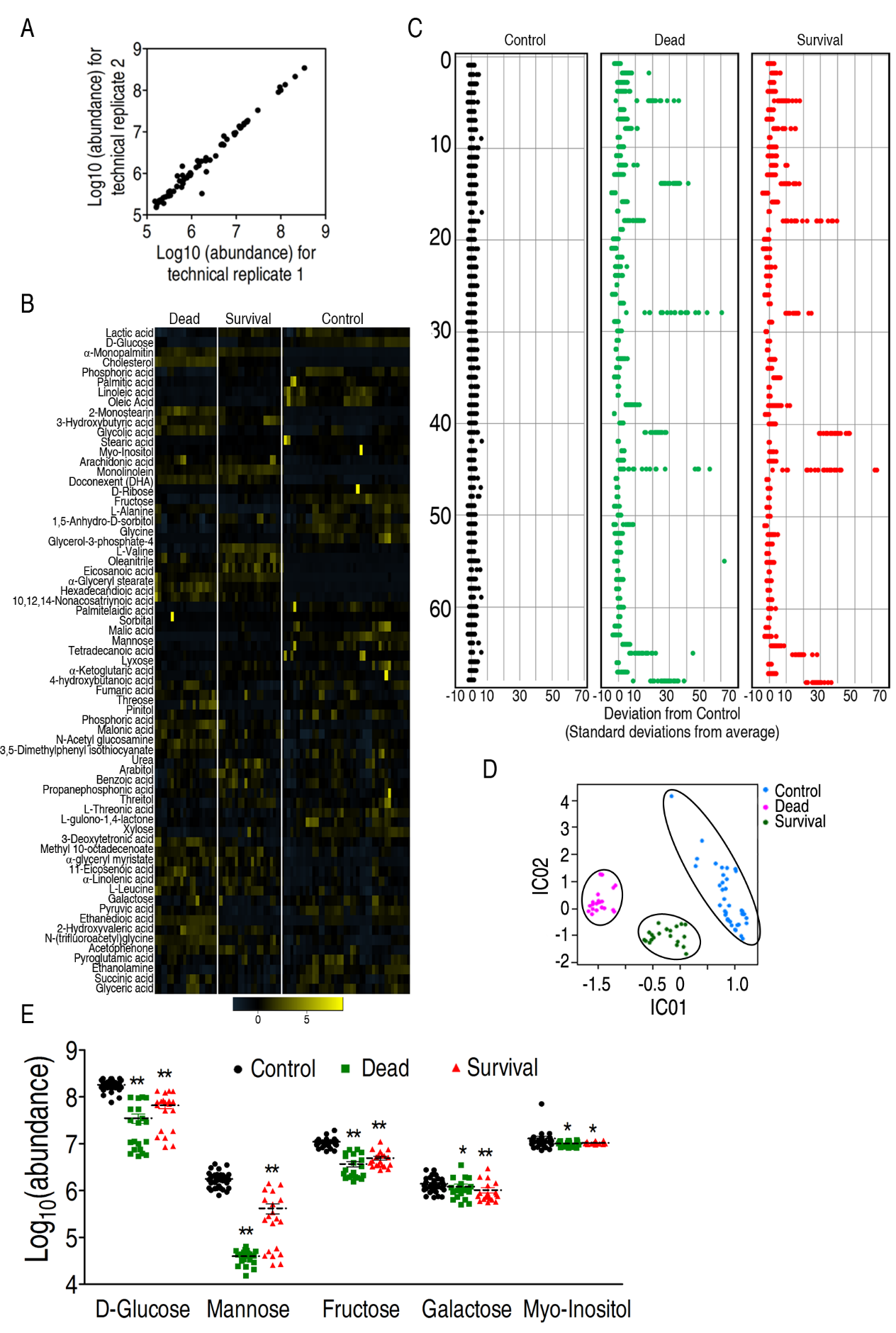
**

**Supplementary Figure 2. Metabolomic analysis of mouse plasma in pre-infection (control), survival and dead groups.** **(A)** Reproducibility of metabolomics profiling platform used in the discovery phase. Spearman correlation coefficient between technical replicates varies between 0.995 and 0.999. **(B)** Representation of 68 metabolites using heat-map. **(C)** *Z*-score plots for metabolite data normalized to the mean of the samples in pre-infection group. (black, control; green, dead; red, survival). **(D)** ICA analysis of metabolite profiles among control, dead and survival groups. **(E)** Abundance of differential metabolites in one of enriched pathway, galactose metabolism. Error bars ± s .e .m，**p*< 0.05，***p*< 0.01. A

B

C D

**Supplementary Figure 3 The L-valine and L-arginine concentrations in mouse serum (A and B) and organs (C and D) were quantified by UPLC-MS.** L-valine (0.5g kg^-1^) or L-valine(0.5g kg^-1^) plus L-arginine (0.25g kg^-1^) or equal volume of sterile saline were intravenously administrated for mice at 0, 3, 6, 9 and 19 h through tail vein. The mouse serums were collected at 0, 5’, 15’, 30’, 1, 4, 7, 10, 20 and 24 h, and liver, kidney and spleen were collected at 24 h after the injection. Error bars ± s .e .m，**p*< 0.05，***p*< 0.01.

**
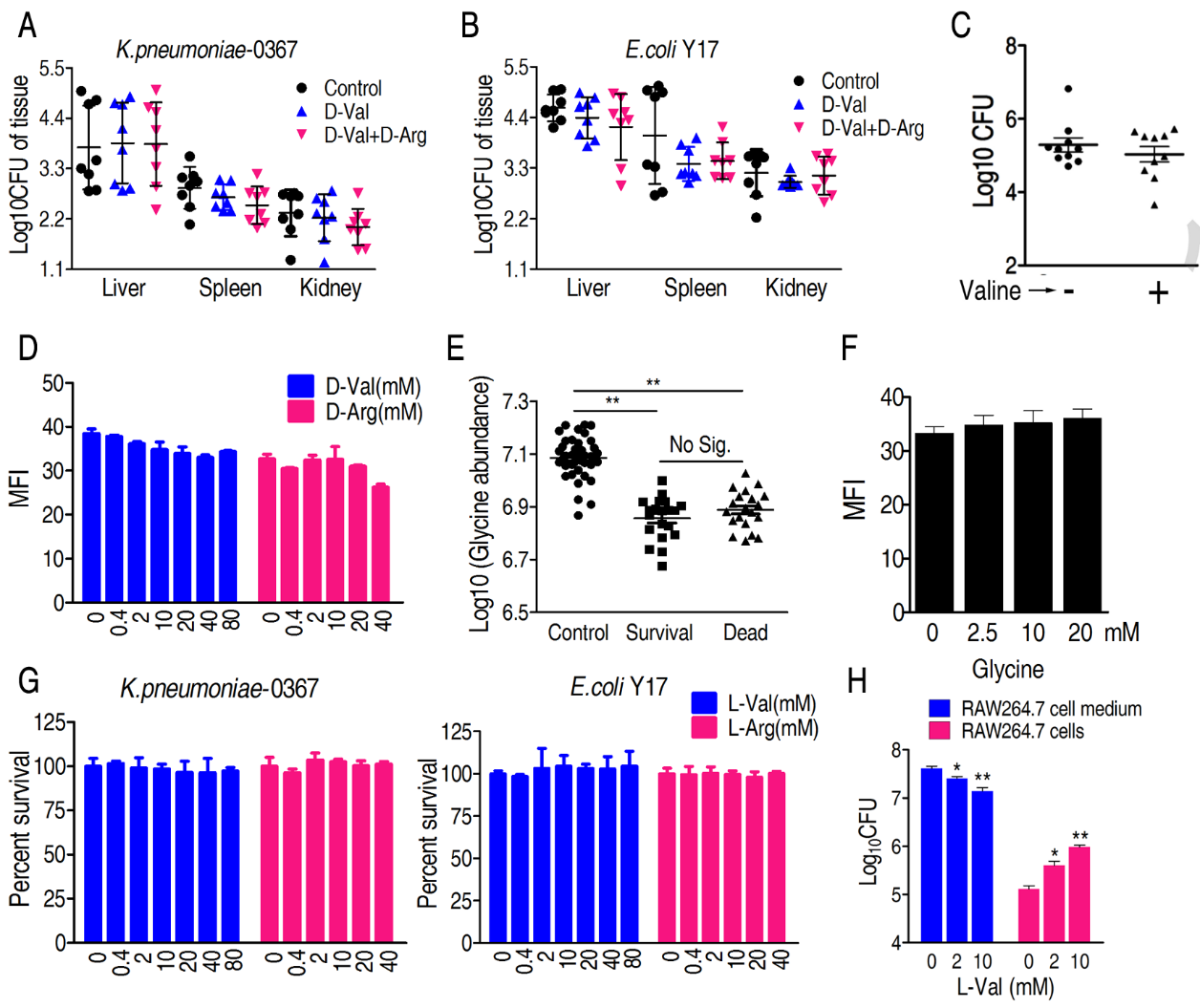
**

**Supplementary Figure 4. Effect of D-valine and D-arginine on bacterial load *in vivo* and phagocytosis in RAW264.7 cells.** D-valine (0.5g kg^-1^) or D-valine plus D-arginine (0.25g kg^-1^) don’t decrease the load of clinical *K. pneumoniae* **(A)** and *E. coli*-Y17 **(B)** in liver, spleen and kindey. **(C)** Effect of plasma on load of *K. pneumoniae* in absence and presence of L-valine. First, L-valine (0.5g kg^-1^) or equal volume of sterile saline are intravenously administrated for mice through tail vein. 2 h later, 100 μL serum are collected from control and L-valine group. Then, equal amounts of *K. pneumoniae* are added into the serum of two groups. After incubation for 24 h, samples are diluted in appropriate multiple, and then plated onto LB medium. **(D)** MFI of GFP-*E. coli* by D-valine or D-arginine-pretreated RAW264.7 cells at the indicated concentrations. **(E)** Abundance of glycine in mouse plasma. **(F)** Effect of glycine on macrophage phagocytosis. **(G)** Percent survival of *K. pneumoniae* and *E. coli*-Y17 in the indicated concentrations of L-valine and L-arginine. Bacteria are re-suspended in sterile saline. Bacterial samples are added to different concentrations of L-valine or L-arginine and incubated at 37 ^o^C for 3 h. After incubation, 100 μL aliquot samples are diluted in appropriate multiple, and then plated onto LB medium. Bacteria are counted when apparent single colony appeares in medium after growing at 37 ^o^C. **(H)** L-Valine stimulations diminish the bacterial counts in cell medium and increase the bacterial counts in cells. *K. pneumoniae* were centrifuged onto macrophages at a multiplicity of infection (MOI) of 100 in the indicated medium. After infection for 1.5 h, macrophages were vigorously washed with 3 mL of cold PBS and harvested in 1 mL of ice-cold PBS. These two fractions of PBS solution were serially diluted in sterile saline, and bacteria were counted when a single colony appeared in the media after growth at 37°C. Error bars ± s .e .m.，**p*< 0.05，***p*< 0.01.
